# LGALS1 was related to the prognosis of clear cell renal cell carcinoma identified by weighted correlation gene network analysis combined with differential gene expression analysis

**DOI:** 10.3389/fgene.2022.1046164

**Published:** 2023-01-12

**Authors:** Jiang Fang, Xinjun Wang, Jun Xie, Xi Zhang, Yiming Xiao, JinKun Li, Guangcheng Luo

**Affiliations:** ^1^ Zhongshan Hospital Xiamen University, School of Medicine, Xiamen University, Xiamen, China; ^2^ Affiliated Hospital of North Sichuan Medical College, Nanchong, Sichuan, China; ^3^ The school of Clinical Medicine, Fujian Medical University, Fuzhou, China

**Keywords:** clear cell renal cell carcinoma, WGCNA, differential gene expression analysis, hub gene, prognosis

## Abstract

Understanding the molecular mechanism of clear cell renal cell carcinoma (ccRCC) is essential for predicting the prognosis and developing new targeted therapies. Our study is to identify hub genes related to ccRCC and to further analyze its prognostic significance. The ccRCC gene expression profiles of GSE46699 from the Gene Expression Omnibus (GEO) database and datasets from the Cancer Genome Atlas Database The Cancer Genome Atlas were used for the Weighted Gene Co-expression Network Analysis (WGCNA) and differential gene expression analysis. We screened out 397 overlapping genes from the four sets of results, and then performed Gene Ontology (GO) enrichment analysis and Kyoto Encyclopedia of Genes and Genome (KEGG) pathways. In addition, the protein-protein interaction (PPI) network of 397 overlapping genes was mapped using the STRING database. We identified ten hub genes (KNG1, TIMP1, ALB, C3, GPC3, VCAN, P4HB, CHGB, LGALS1, EGF) using the CytoHubba plugin of Cytoscape based on the Maximal Clique Centrality (MCC) score. According to Kaplan-Meier survival analysis, higher expression of LGALS1 and TIMP1 was related to poorer overall survival (OS) in patients with ccRCC. Univariate and multivariate Cox proportional hazard analysis showed that the expression of LGALS1 was an independent risk factor for poor prognosis. Moreover, the higher the clinical grade and stage of ccRCC, the higher the expression of LGALS1. LGALS1 may play an important role in developing ccRCC and may be potential a biomarker for prognosis and treatment targets.

## Introduction

Kidney cancer is one of the most common tumors, accounting for 5% and 3% of all adult malignancies in men and women, respectively, ranking sixth among men and eighth among women (Siegel et al., 2020). Renal cell carcinoma (RCC) denotes cancer originating from the renal epithelium, accounting for more than 90% of cancers in the kidney. Clear cell renal cell carcinoma (ccRCC) is the most common tumor in RCC and the cause of most cancer-related deaths (Hsieh et al., 2017). Radiotherapy and chemotherapy are not effective for ccRCC, and surgery is the first choice for early and locally advanced ccRCC, while targeted therapies and immunotherapy are the mainstays for advanced ccRCC, including Pazopanib, Sorafenib, Tivozanib, Nivolumab, Ipilimumab, Sunitinib. (Sternberg et al., 2010; Motzer et al., 2013; Motzer et al., 2018). However, there are no available biomarkers for the prognosis and treatment of the disease at present.

Due to the recent development of high-throughput sequencing technology, genomic microarrays, and bioinformatics, bioinformatics analysis has become a new way to reveal the pathogenesis of the disease. Weighted gene expression network analysis (WGCNA) is a systems biology method used to describe the correlation patterns of genes in microarray or RNA Sequence data. And it is an algorithm for discovering highly related gene clusters (modules) and identifying phenotypic-related modules or gene clusters (Langfelder and Horvath 2008). The hub genes and prognostic biomarkers of various cancers, including cholangiocarcinoma (Long et al., 2021), hepatocellular carcinoma (Zhang et al., 2020), and endometrial cancer (Liu et al., 2019), were identified by WGCNA.

In addition, differential gene expression analysis is another powerful analysis in transcriptomics that determines genotypic differences between two or more cell conditions to support specific hypothesis-driven research, and is one of the most common applications of RNA sequencing (RNA-seq) data and may reveal potential biomarkers of disease (McDermaid et al., 2019). Hence, the results of differential gene expression analysis and WGCNA were combined for further analysis, which can improve the recognition ability of highly related genes. The final screened genes can be used as candidate biomarkers.

**FIGURE 1:**
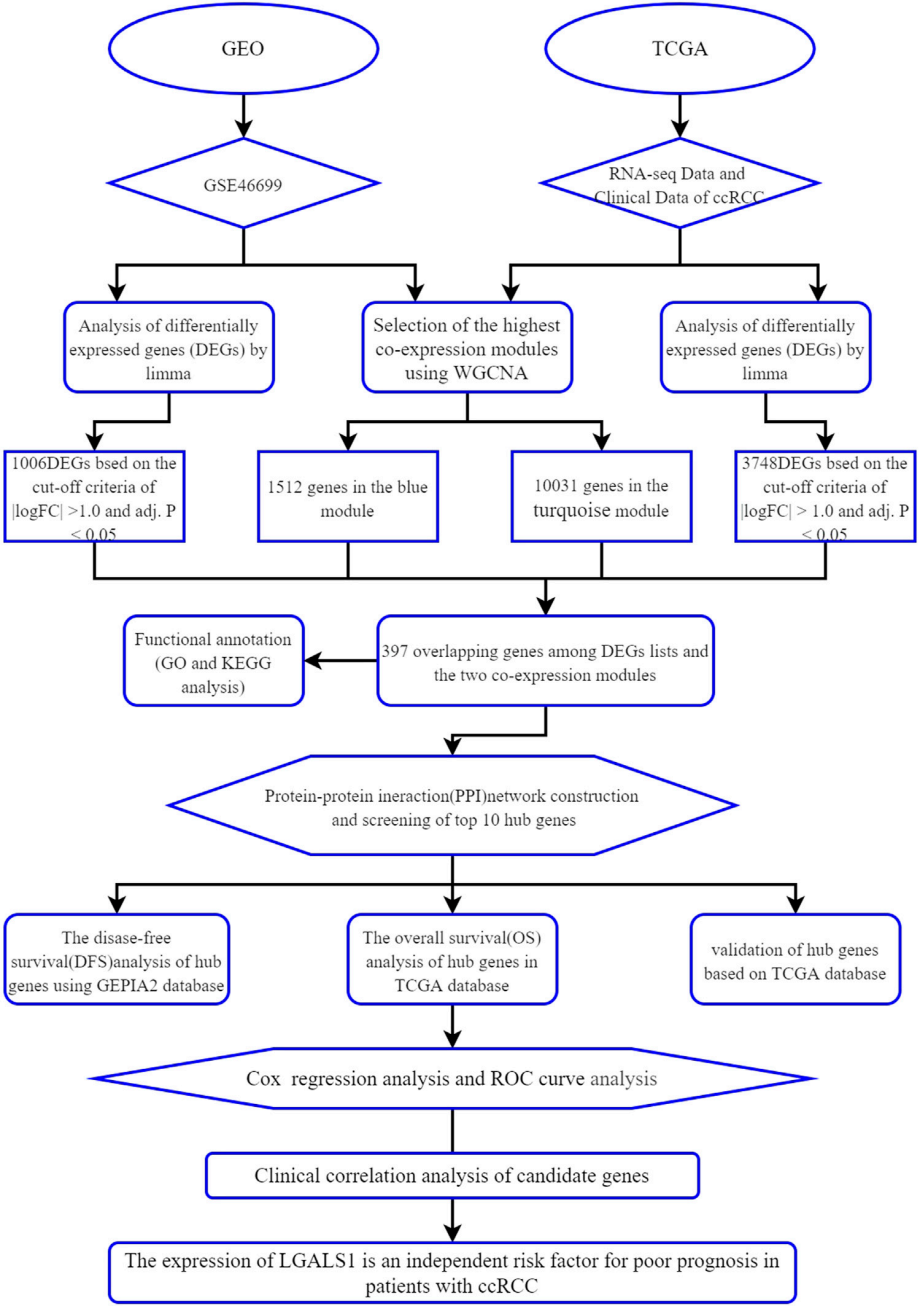
Workflow of this study.

In our study, differential gene expression analysis and WGCNA were performed to identify differentially co-expressed genes by using the mRNA expression data of ccRCC from the Gene Expression Omnibus (GEO) and The Cancer Genome Atlas (TCGA) database. GO enrichment analysis, KEGG pathway analysis, and protein-protein interaction (PPI) analysis combined with survival analysis were used to explore the development of ccRCC. Moreover, the reliability of hub genes was verified and the clinical correlation of meaningful genes was analyzed. As far as we know, this is the first time to use the data from the GEO and TCGA database for WGCNA combined with differential gene expression analysis to screen out the key genes related to ccRCC and to establish a survival model to predict the prognosis of ccRCC. The workflow of this study is displayed in Figure 1.

## Materials and methods

### Download and preprocess data from the GEO and TCGA databases

The Gene Expression Omnibus (GEO; https://www.ncbi.nlm.nih.gov/geo/) and the Cancer Genome Atlas (TCGA; https://portal.gdc.cancer.gov/) are publicly available cancer databases. Download the microarray data of ccRCC from the GEO database and the RNA-seq data and clinical information of ccRCC from the TCGA database, Firstly, we downloaded the normalized gene expression matrix files and platform annotations of the GSE46699 dataset from the GEO database. GSE46699 was composed of 66 tumor tissues and 64 normal tissues from the ccRCC samples. Then, we used the platform annotation file to convert the probe into gene symbols and removed the repeated probes of the same gene by determining the median expression value of all the corresponding probes. In consequence, a total of 21654 genes were chosen for further analysis.

The RNA sequencing (RNA-Seq) expression data of 611 samples and the corresponding clinical information of 537 cases were downloaded from the TCGA database. Among the 611 samples, 72 cases were normal and 539 cases were ccRCC. The *edgeR* R package tutorial recommends that genes with low expression do not need to be further analyzed (Robinson et al., 2010). Therefore, we used CPM (count per million) to correct the data and analyze the genes with CPM >1. We used the rpkm function in the *edgeR* R package to select the data by dividing the gene count by the gene length, then the rpkm values were output. Finally, 14684 genes with RPKM values were obtained for the next analysis.

## Identify key co-expression modules by WGCNA

In our research, the R package termed *WGCNA* was used to construct a gene co-expression network based on the gene expression data profiles of GSE46699 and TCGA-KIRC (Langfelder and Horvath 2008). WGCNA was used to find the modules with the most significant differences between normal and tumor samples and to extract genes from the modules. An adjacency matrix was constructed to describe the correlation strength between nodes. The formula of the adjacency matrix was as follows:

$$sij=\left|cor\left(xi,\;xj\right)\right|aij={Sij}^{\beta }$$

where *i* and *j* represented two different genes, and *Xi* and *Xj* were their expression values, respectively. *sij* represented Pearson’s correlation coefficient, and *aij* represented the strength of the correlation between two genes. *β* was a soft threshold, which was the Pearson correlation coefficient *β* of each pair of genes. Pearson correlation matrix was transformed into an adjacency matrix (scale-free network) by a *β*-power operation. In this study, we chose the soft power *β* = 16 and 2. Then, we converted the adjacency matrix into a topological overlap matrix (TOM) and the corresponding degree of dissimilarity (1-TOM). Subsequently, the hierarchical clustering dendrogram of the 1-TOM matrix was constructed, and the similar gene expression was divided into different gene co-expression modules, each module contained at least 50 genes. According to the previous research (Li et al., 2019), we calculated the module-trait correlation between the modules and clinical trait information to further determine the functional modules in the co-expression network. The method of dynamic tree cutting was used to identify modules from the hierarchical clustering tree, and the module eigengene (ME) of each module was calculated. ME represented the overall expression level of the module. Finally, the module with a high correlation coefficient was considered as a candidate module related to clinical traits and was chosen for follow-up analysis.

### Screening the differentially expressed genes (DEGs)

In this study, the *limma* R package was used to screen the differentially expressed genes (DEGs) between ccRCC and normal kidney tissue in the data downloaded from the GEO and TCGA, respectively (Ritchie et al., 2015), using the Benjamini–Hochberg method to adjust the *p*-value to control the false discovery rate (FDR). The adj. *p* <0.05 and |logFC| >1.0 were selected as cut-off criteria for DEGs. Volcano plots were drawn using the *ggplot2* R package in R version 4.03.

### Intersecting the genes in the module of interest

The overlapping genes, obtained by intersecting the DEGs with the co-expression genes extracted from the co-expression network, were used to determine potential prognostic genes. Use the *VennDiagram* R package in R software to map the genes we obtained into a Venn diagram (Chen and Boutros 2011).

### Functional annotation and pathway enrichment analysis

The *clusterProfiler* R package was used to visualize gene ontology (GO) enrichment analysis and Kyoto Encyclopedia of Genes and Genomes (KEGG) pathway of key genes (Yu et al., 2012). The cut-off criterion was set at adjusted *p* <0.05.

### PPI network construction and hub genes identification

The key genes were imported into the *STRING* online database (https://string-db.org/) (Szklarczyk et al., 2019). Genes with a confidence score ≥.9 were chosen to build a protein-protein interaction (PPI) network model and visualized by Cytoscape (v3.8.2) (Shannon et al., 2003). A Cytoscape plugin, cytoHubba, can be used to rank nodes in a network by their network features. Among the eleven methods to find hub nodes, Maximal Clique Centrality (MCC) was the most effective one. The MCC of each node was calculated by CytoHubba (Chin et al., 2014), and the genes with the top 10 MCC scores were identified as hub genes for this study.

### Hub genes validation

To verify the reliability of the hub genes, the differentially expressed genes (DEGs) of hub genes obtained from the TCGA database were tested by Wilcoxon test in R software using the *limma* R package (Ritchie et al., 2015), and the expression level of each hub gene between normal tissues and tumor tissues was visualized as a boxplot. Set the cut-off criterion of DEGs to *p* <0.05.

### Verification of hub genes with survival analysis

To validate whether the survival of ccRCC patients was affected by hub genes, the data obtained from the TCGA, including the clinical data and gene expression data of 530 ccRCC samples, were used to seek the relationship between hub genes and Overall survival (OS) in patients by Kaplan-Meier univariate survival analysis with the survival R package in R software. In addition, using the online tool GEPIA2 (http://gepia2.cancer-pku.cn/#index) to determine the relationship between hub genes expressed in ccRCC patients and Disease-free survival (DFS) (Tang et al., 2019). The log-rank *p* <0.05 of the survival-related hub genes was considered statistically significant.

### Construction of the prognostic risk model

Cox regression analysis was used to construct the prognostic risk model of ccRCC, and the *survival* package in R was used for univariate and multivariate Cox proportional hazard regression analysis. The Cox analysis signature included age, gender, grade as well stages. The sensitivity and specificity of the Receiver Operation characteristic (ROC) curve were used to evaluate the prognostic value of the signature. All analyses were performed using R.

### Correlation between hub genes with the clinicopathological characters

Clinicopathological data of ccRCC patients, including gender, Grade, and tumor stage were collected from the TCGA database. We conducted a correlation analysis between hub genes and clinical traits. The analysis was conducted by chi-square test under R environment. And the *ggplot2* R package was used to draw boxplots. The criterion for statistical significance was set as *p* <0.05.

## Results

### Construction of weighted gene Co-expression and division of modules

**FIGURE 2:**
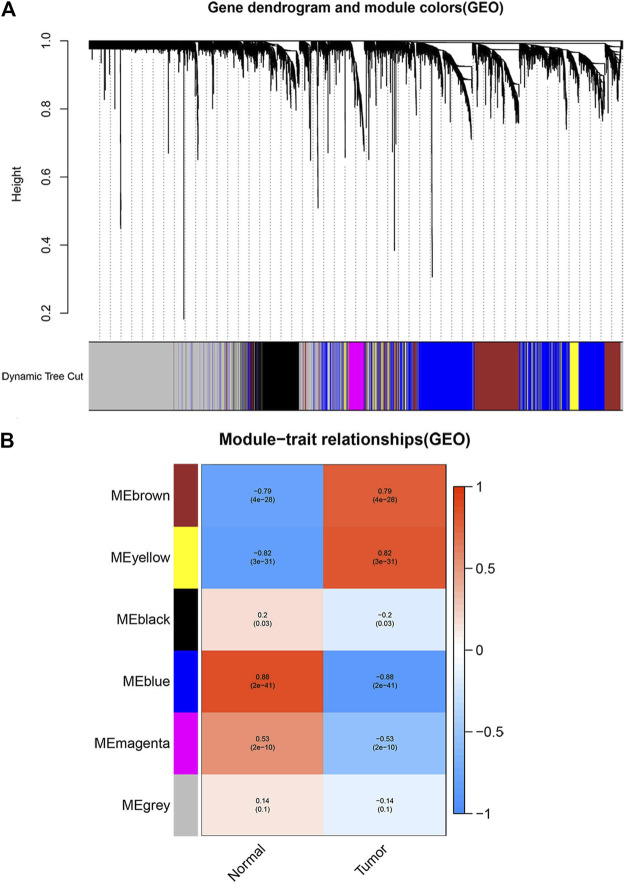
Screen the modules correlated with the clinical information from the GSE46699 dataset. **(A)** The hierarchical clustering of genes based on the 1-TOM matrix is used to sort the clustering tree of co-expression network modules. Mark each module with a different color. **(B)** Module-trait relationship in GSE46699. Each row corresponds to a color module, and each column corresponds to the tumor and normal. Each cell consists of the corresponding *p*-value and correlation.

**FIGURE 3:**
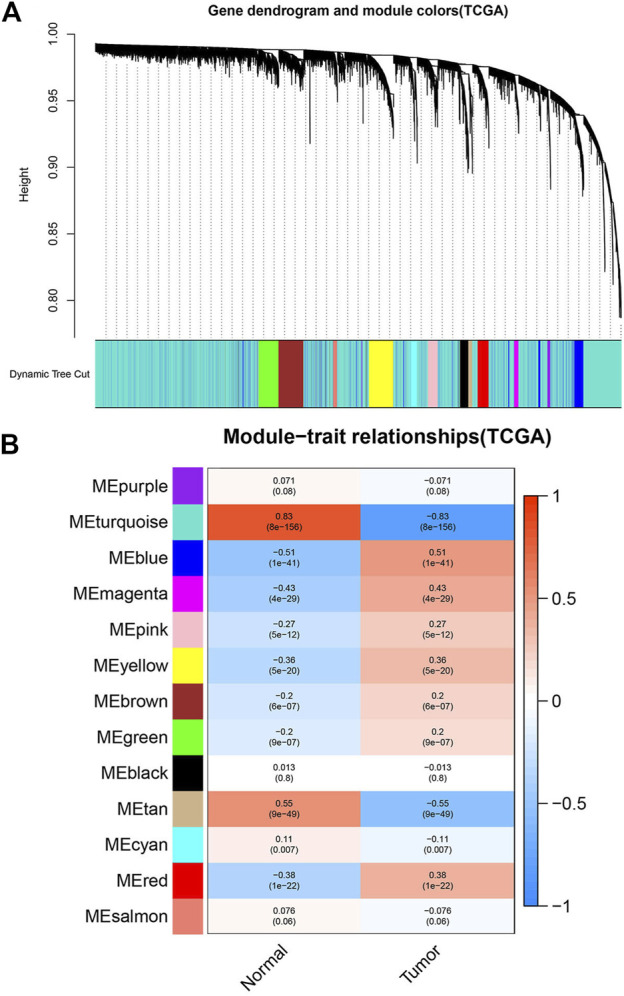
Screen the modules correlated with the clinical information from the TCGA- KIRC dataset. **(A)** The hierarchical clustering of genes based on the 1-TOM matrix is used to sort the clustering tree of co-expression network modules. Each module is assigned a different color. **(B)** Module-trait relationship in TCGA- KIRC. Each row corresponds to a color module, and each column corresponds to the tumor and normal. Each cell consists of the corresponding *p*-value and correlation.

The *WGCNA* R package was used to construct the Weighted gene co-expression networks from the GSE46699 and TCGA- KIRC datasets for finding the functional clusters of ccRCC patients. The screened genes in the GSE46699 dataset were divided into five cox-expression modules (Figure 2A) and the screened genes in TCGA- KIRC were divided into 13 cox-expression modules (Figure 3A) (excluding the grey module, the genes of this gene set were not clustered into any module), and each module was assigned a color. After that, to assess the correlation between two clinical traits (normal and tumor) and each module, we drew the module-traits relationships heatmap. Figure 2B; Figure 3B showed the module-trait relationships. The blue module of the GSE46699 dataset and the turquoise module of TCGA- KIRC have the highest correlation with normal tissues (blue module: *r* = .88, *p* = 2e−41; turquoise module: *r* = .83, *p* = 8e-156), the blue module of the GSE46699 and the turquoise module of TCGA-KIRC included 1,512 and 10031 co-expression genes, respectively.

### DEGs screening and identification of common genes between co-expression modules and DEGs

**FIGURE 4:**
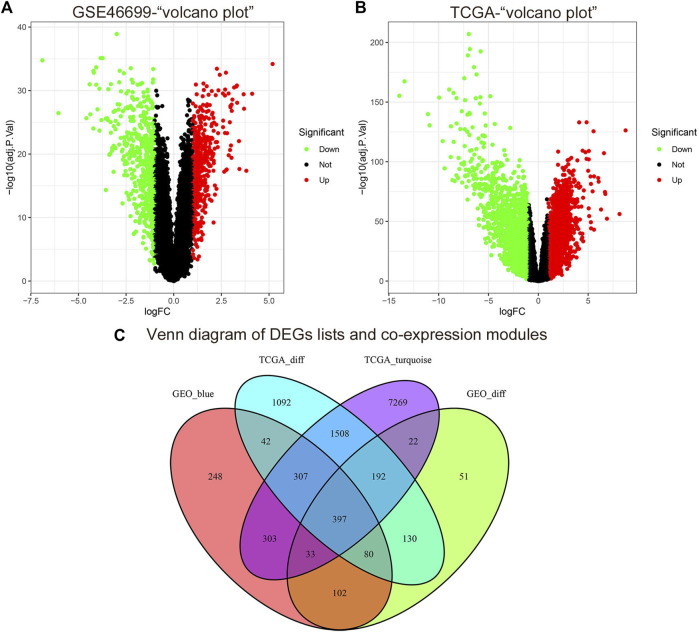
The cut-off criteria for screening differentially expressed genes in GSE46699 and TCGA datasets of ccRCC were | log FC |> 1.0 and adj. *p* <0.05. **(A)** Volcano map of differentially expressed genes in the GSE46699 data set. **(B)** Volcano map of differentially expressed genes in the TCGA dataset. **(C)** Venn diagram of gene crossover between differentially expressed genes and co-expression module. In total, 397 overlapping genes in the intersection of differentially expressed genes and two co-expression modules.

According to the cut-off criteria of |logFC| ≥ 1.0 and adj. *p* < .05, we used the *limma* R package to find that 1,007 DEGs in the GSE46699 dataset (Figure 4A) and 3,748 DEGs in TCGA-KIRC (Figure 4B) were abnormally regulated in tumor tissues. Then, we got a Venn diagram using the *VennDiagram* R package (Figure 4C). In total, 397 overlapping genes were extracted for further study.

### Enrichment analysis of GO and KEGG

**FIGURE 5:**
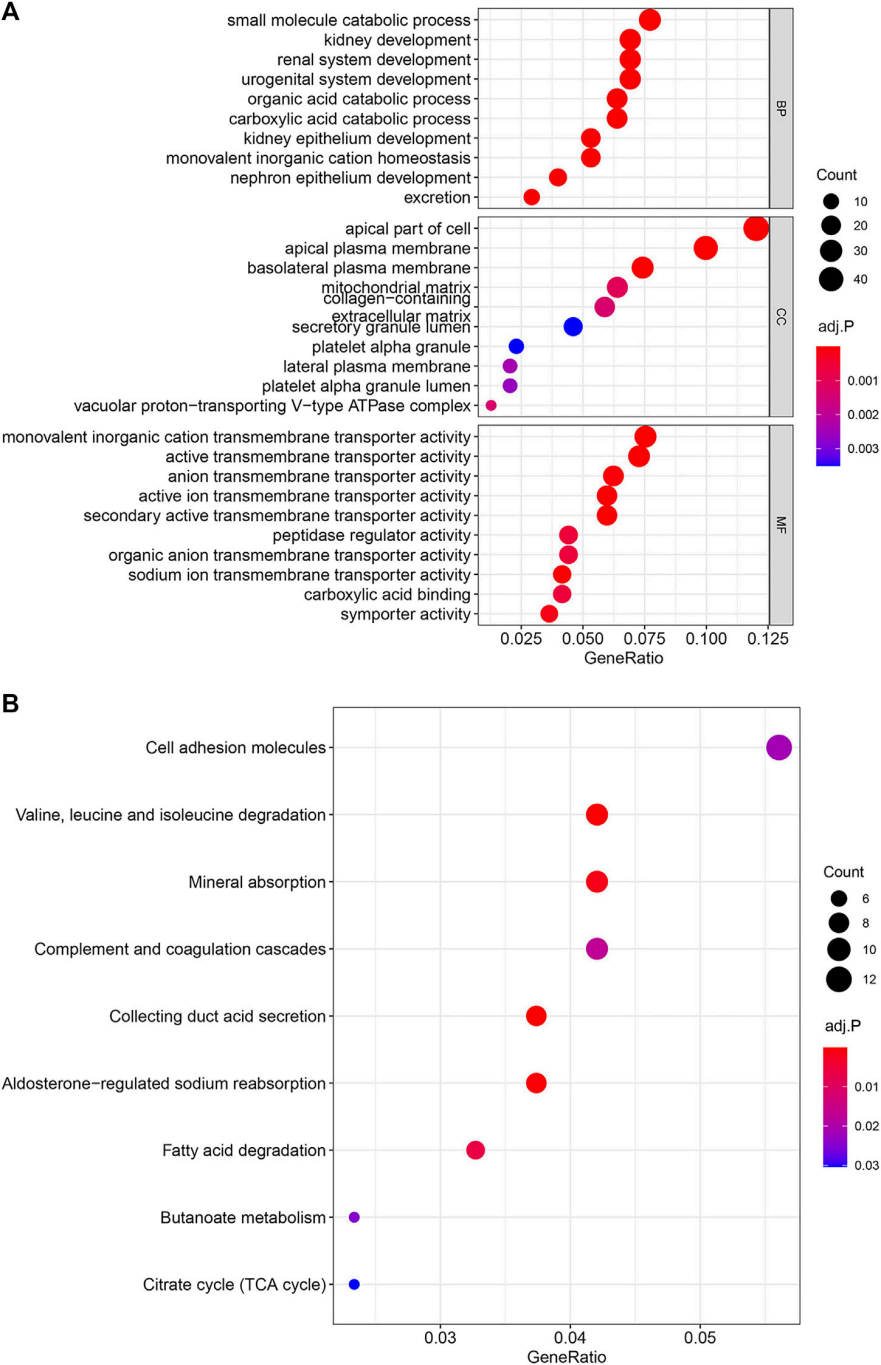
Enrichment Analysis of GO and KEGG. The color represents the adjusted *p*-values, and the size of the spots represents the gene number. **(A)** Gene Ontology (GO) analysis of the 397 overlapping genes. **(B)** KEGG analysis of the 397 overlapping genes.

To explore the biological processes and signal pathways of the main enrichment of these 397 genes, the *clusterProfiler* R package was used for GO and KEGG analysis. The GO enrichment analysis includes three parts: molecular function (MF), biological process (BP), and cell component (CC). GO analysis revealed that the most significant molecular function (MF), biological process (BP), and cell component (CC) were monovalent inorganic cation transmembrane transporter activity, small molecule catabolic process, and apical part of cell, respectively (Figure 5A). And KEGG analysis showed the signaling pathway of key genes was mostly related to the Cell adhesion molecules (Figure 5B).

### Construction of PPI network and identification of hub genes

**FIGURE 6:**
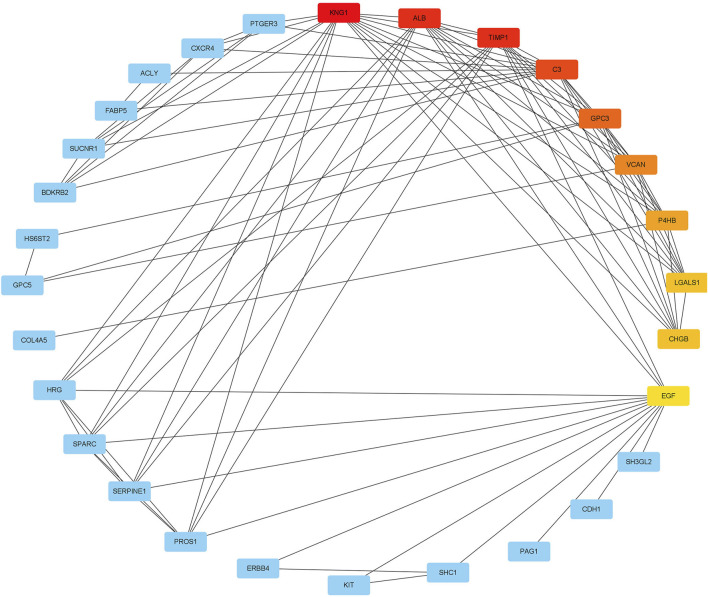
The maximal clique centrality (MCC) algorithm was used to identify the hub genes in PPI networks. The edges represented the connection between proteins and proteins. The red nodes represented the genes with high MCC scores, while the yellow node represented the genes with low MCC scores.

We used the *STRING* database to construct a PPI network between overlapping genes. Then, the MCC algorithm of the CytoHubba plugin was used to select the hub genes from the PPI network, which was plotted in Figure 6. According to the MCC scores, the genes with the top ten highest scores were determined as hub genes, including LGALS1, TIMP1, KNG1, ALB, C3, GPC3, VCAN, P4HB, CHGB, and EGF.

### Validation and survival analysis of hub genes

**FIGURE 7:**
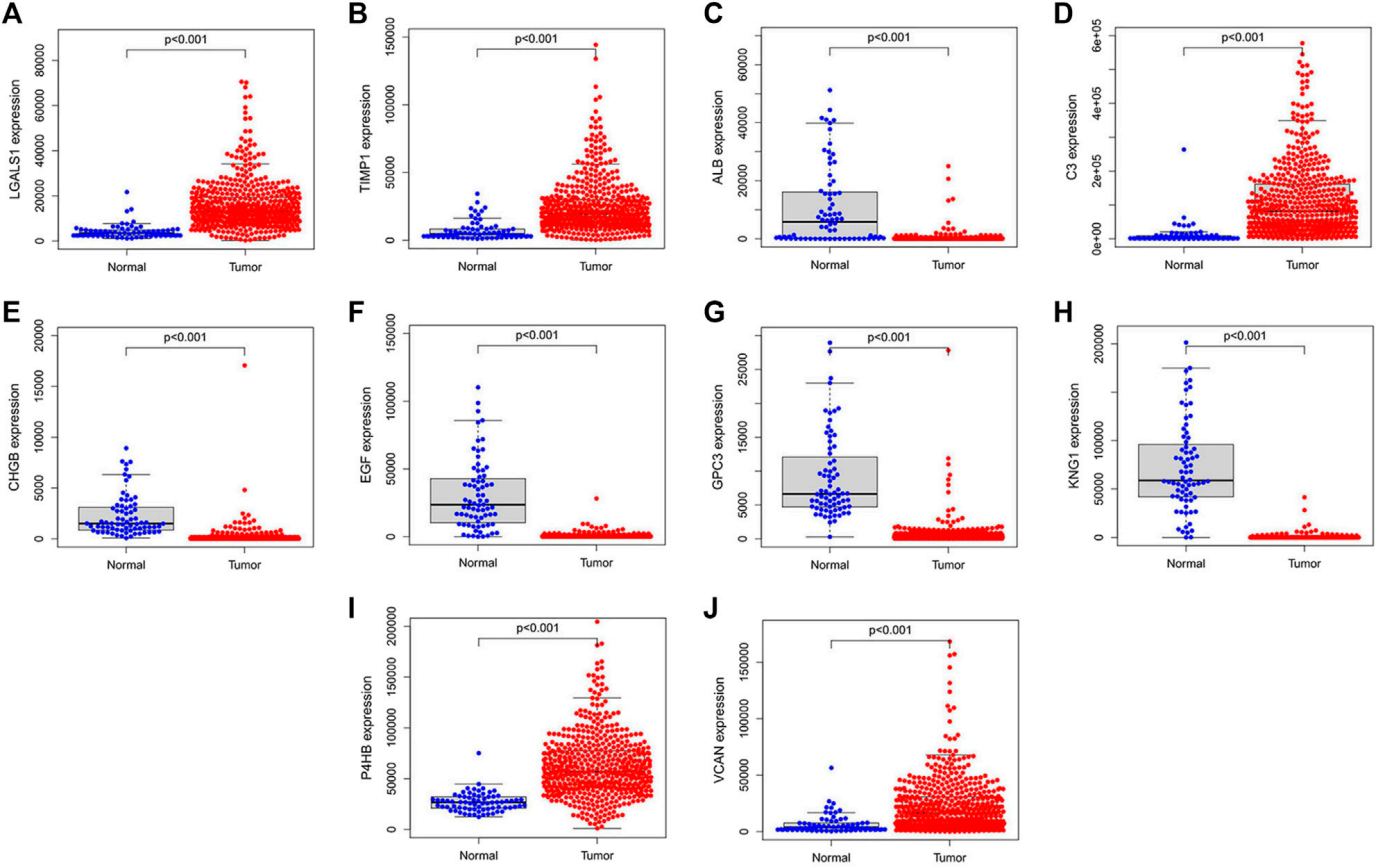
Hub genes validation using TCGA dataset. **(A)** The expression of LGALS1 in tumor and normal tissues in patients with ccRCC. **(B)** The expression of TIMP1 in tumor and normal tissues in patients with ccRCC. **(C)** The expression of ALB in tumor and normal tissues in patients with ccRCC. **(D)** The expression of C3 in tumor and normal tissues in patients with ccRCC. **(E)** The expression of CHGB in tumor and normal tissues in patients with ccRCC. **(F)** The expression of EGF in tumor and normal tissues in patients with ccRCC. **(G)** The expression of GPC3 in tumor and normal tissues in patients with ccRCC. **(H)** The expression of KNG1 in tumor and normal tissues in patients with ccRCC. **(I)** The expression of P4HB in tumor and normal tissues in patients with ccRCC. **(J)** The expression of VCNA in tumor and normal tissues in patients with ccRCC.

**FIGURE 8:**
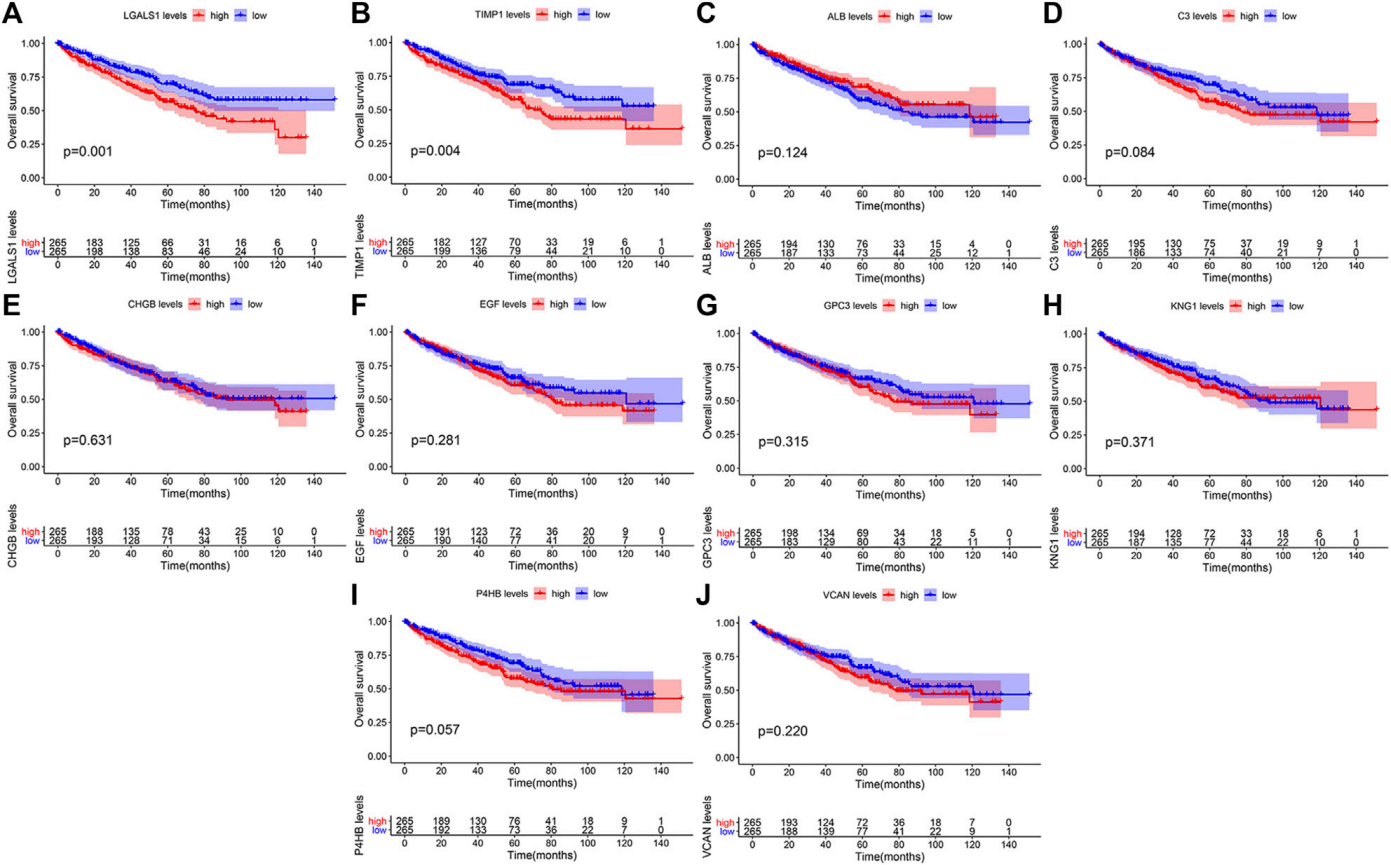
Overall survival (OS) of the ten hub genes in patients with ccRCC according to the Kaplan-Meier survival analysis. The patients were divided into high-expression and low-expression groups based on the median expression. **(A)** Survival analysis for LGALS1 in ccRCC. **(B)** Survival analysis for TIMP1 in ccRCC. **(C)** Survival analysis for ALB in ccRCC. **(D)** Survival analysis for C3 in ccRCC. **(E)** Survival analysis for CHGB in ccRCC. **(F)** Survival analysis for EGF in ccRCC. **(G)** Survival analysis for GPC3 in ccRCC. **(H)** Survival analysis for KNG1 in ccRCC. **(I)** Survival analysis for P4HB in ccRCC. **(J)** Survival analysis for VCNA in ccRCC.

**FIGURE 9:**
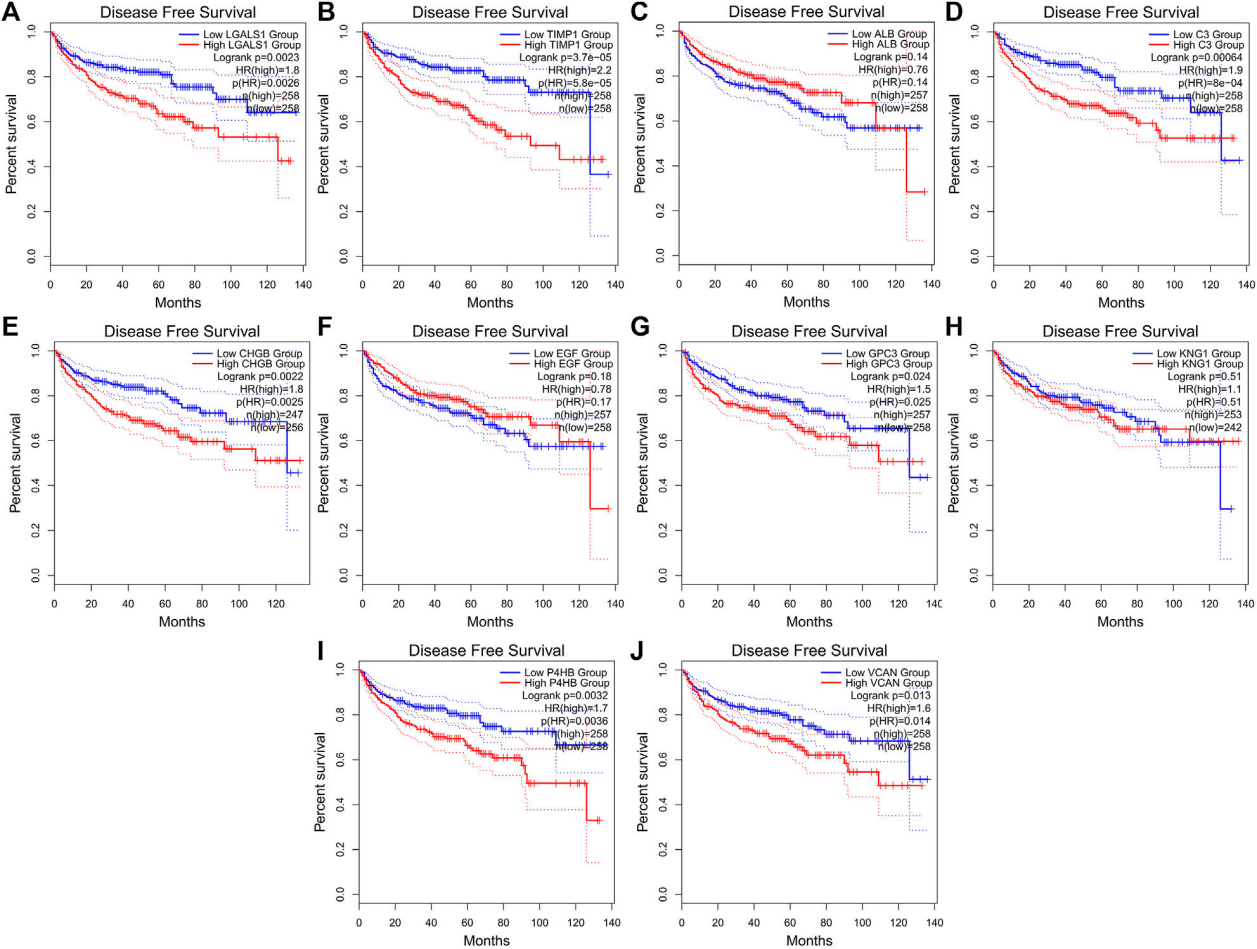
Disease-free survival (DFS) analysis of the ten hub genes of ccRCC patients in the GEPIA2 database. **(A)** Survival analysis for LGALS1 in ccRCC. **(B)** Survival analysis for TIMP1 in ccRCC. **(C)** Survival analysis for ALB in ccRCC. **(D)** Survival analysis for C3 in ccRCC. **(E)** Survival analysis for CHGB in ccRCC. **(F)** Survival analysis for EGF in ccRCC. **(G)** Survival analysis for GPC3 in ccRCC. **(H)** Survival analysis for KNG1 in ccRCC. **(I)** Survival analysis for P4HB in ccRCC. **(J)** Survival analysis for VCNA in ccRCC.

We validated the expression levels of hub genes among the patients of the TCGA-KIRC dataset, which was displayed in Figure 7. We found that ten hub genes in ccRCC up-regulated significantly more than that in normal tissues. Then, we used the *survival* R package and GEPIA2 database to do OS and DFS analysis on ten hub genes through the Kaplan-Meier curve, to explore prognostic values of hub genes in ccRCC patients. As shown in Figure 8, the results illustrated that LGALS1 and TIMP1 were significantly associated with the overall survival of the ccRCC patients (*p* <0.05), and higher expression of LGALS1 and TIMP1 was related to poorer overall survival (OS) in patients with ccRCC. And Disease-Free survival in patients with ccRCC was associated with the expression levels of LGALS1, TIMP1, C3, CHGB, GPC3, P4HB, and VCAN (*p* < .05) (Figure 9), high levels of LGALS1, TIMP1, C3, CHGB, GPC3, P4HB, and VCAN were correlated with low Disease-Free survival in patients with ccRCC.

### Verification of prognostic model

**FIGURE 10:**
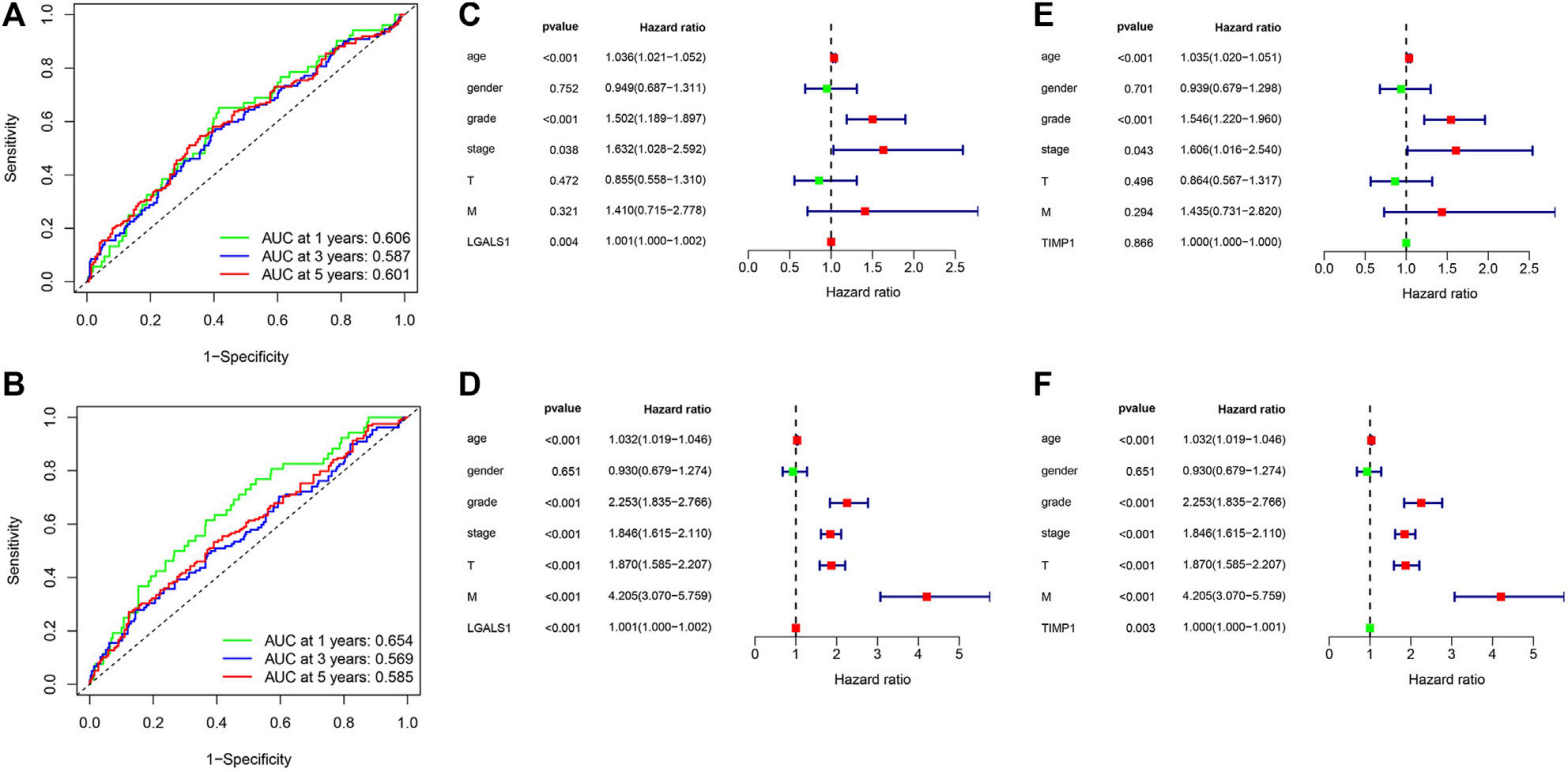
**(A)** ROC curve for LGALS1 in ccRCC patients. **(B)** ROC curve for TIMP1 in ccRCC patients. **(C)** Univariate Cox regression analysis of LGALS1 expression and clinicopathologic characteristics. **(D)** multivariate Cox regression analysis of LGALS1 expression and clinicopathologic characteristics. **(E)** Univariate Cox regression analysis of TIMP1 expression and clinicopathologic characteristics. **(F)** multivariate Cox regression analysis of TIMP1 expression and clinicopathologic characteristics.

Univariate regression analysis showed that the expression of LGALS1 and TIMP1, age, grade, and stage were related to the prognosis of ccRCC (Figures 10D, F). Multivariate regression analysis showed that the expression of LGALS1, age, grade and stage were independent risk factors correlated with the prognosis of ccRCC patients (Figures 10C, E). To evaluate the prognostic validity of the risk score, the ROC curve showed that the AUCs of 1-year, 3-year, and 5-year survival expressed by LGALS1 were .606, .587 and .601, respectively, while the expression of TIMP1 were .654, .569, and .585, respectively, indicating that the risk model was significantly effective and applicable.

### Clinical correlation analysis of survival-related genes

**FIGURE 11:**
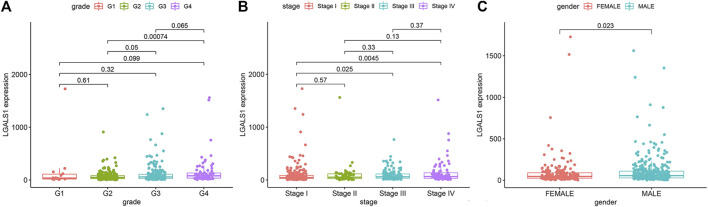
**(A)** Boxplot of the association between the expression of LGALS1 and Grade. **(B)** Boxplot of the association between the expression of LGALS1 and Stage. **(C)** Boxplot of the association between the expression of LGALS1 and Gender.

We analyzed the correlation between the expression of LGALS1 and clinical data downloaded from the TCGA database (Table 1). As shown in Figure 11, there were significant differences in the expression of LGALS1 from Grade 2 to Grade 3 (*p* = 0.05) and Grade 2 to Grade 4 (*p* = 0.00074). In addition, there were significant differences in the expression of LGALS1 from Stage I to Stage III (*p* = 0.025) and Stage I to Stage IV (*p* =0.0045). Moreover, there are differences in the expression of LGALS1 (*p* =0.023) between males and females in ccRCC patients. These results showed that the higher the clinical grade and stage of ccRCC, the higher the expression of LGALS1. And LGALS1 was highly expressed in male patients with ccRCC.

**TABLE 1:** Clinical data clinical correlation analysis of survival related genes of TCGA-KIRC.

			Sample number	
Gender				
	Female		191	
	Male		346	
Grade				
	G1		14	
	G2		230	
	G3		207	
	G4		78	
	GX		5	
	unknow		3	
Stage	Stage I		269	
	Stage II		57	
	Stage III		125	
	Stage IV		83	
	unknow		3	

## Discussion

For kidney cancer, about one-third of patients were diagnosed with regional or distant metastases. The 5-Year Relative Survival of patients with distant metastasis was only 13.9%. Radical nephrectomy is the standard treatment for localized primary kidney cancer, but approximately one-quarter of these have relapses in distant sites (Choueiri and Motzer 2017). With the development of bioinformatics, although some biomarkers related to ccRCC have been found in recent years, such as AOX1 (Xiong et al., 2021), circCHST15 (Gui et al., 2021), DDX39 (Bao et al., 2021) and PPAR α (Luo et al., 2019), the risk of death in patients with ccRCC is still high, so it is necessary to find more reliable markers and obtain more treatments to reduce the risk of death in patients with ccRCC. We identified 397 differentially expressed genes with consistent expression trends in the GSE46699 and TCGA-KIRC databases by WGCNA and Differential gene expression analysis. Furthermore, we analyzed the KEGG pathway and GO enrichment analysis of the 397 genes, and found that they play a significant role in many biological processes. Based on the MCC scores of the CytoHubba plugin in Cytoscape, the top 10 hub genes correlated with ccRCC were picked out, including, KNG1, TIMP1, ALB, C3, GPC3, VCAN, P4HB, CHGB, LGALS1, and EGF. And all ten hub genes up-regulated significantly in ccRCC compared with the normal tissues. Among ten hub genes, the high expression of LGALS1 and TIMP1 were correlated significantly with the poor OS, and the high expression of LGALS1, TIMP1, C3, CHGB, GPC3, P4HB, and VCAN were correlated with the low DFS of ccRCC significantly. Univariate and multivariate Cox proportional hazard analysis showed that LGALS1 expression was an independent risk factor for poor prognosis. Finally, the correlation between the expression of LGALS1 and clinicopathological features was analyzed.

In the Genome database, the gene that encodes galectin-1 is named LGALS1 (Barondes et al., 1994). Galectin-1 is a homodimer composed of subunits of approximately 130 amino acids, abundant in skeletal, smooth, and cardiac muscle, motor and sensory neurons, thymus, kidney, and placenta. And the carbohydrate recognition domain (CRD) is responsible for β-galactoside binding (Barondes et al., 1994). Galectins play important roles in metastasis, angiogenesis, tumor immunity, proliferation, and apoptosis (Barondes et al., 1994; Méndez-Huergo et al., 2017; Wdowiak et al., 2018; Huang et al., 2021). Galectin-1 can influence the proliferation of CD8†T cells and the immunosuppressive capacity of CD8†CD122†PD-1†Tregs, lower Galectin-1 expression result in reduced tumor growth (Cagnoni et al., 2021). Galectin-1 can interact with oncogenic RAS protein on the cell surface, affect the proliferation of tumor cells through the RAS pathway (Paz et al., 2001; Shih et al., 2019), and promote tumor progression and chemotherapy resistance by up-regulating p38 MAPK, ERK, and cyclooxygenase-2 (Chung et al., 2012), It can also promote tumor invasion and metastasis by activating the FAK/PI3K/AKT/mTOR pathway (Su et al., 2020). In addition, Galectin-1 can affect tumorigenesis by activating the Hh signal pathway (Martínez-Bosch et al., 2014). Galectin-1 is a multifunctional target during Pancreatic ductal adenocarcinoma progression (Orozco et al., 2018). Up-regulation of LGALS1 expression can not only promote the occurrence and development of non-small cell lung cancer cells (Sun et al., 2022), but also promote the proliferation and cell cycle progression of esophageal squamous cell carcinoma cells (Cui et al., 2022). The high expression of Galectin-1 is associated with the migration and invasion of gastric cancer cells and poor prognosis of patients with prostate cancer (Shih et al., 2018) and hepatocellular carcinoma (Tsai et al., 2022). LGALS1 affects the occurrence and development of tumors in many ways, and its high expression is related to the poor prognosis of tumors, which is consistent with our result in ccRCC. Olena Masui et al. verified the abnormal protein expression of Galectin-1 in metastatic and primary renal cell carcinoma by Western blot and immunohistochemical analysis (Masui et al., 2013). N M A White et al. showed that Galectin-1 is a downstream effector molecule of miR-22 and participates in the HIF/mTOR signal axis in renal cell carcinoma (White et al., 2014). In addition, some of their findings were completely consistent with our study, including LGALS1 mRNA expression in ccRCC is significantly higher than normal kidney tissue of the same patient, patients with high LGALS1 expression are correlated with poor overall survival and disease-free survival, and compared with low-grade (grade I or II) tumors, high expression levels of LGALS1 are correlated with high-grade tumors (grade III or IV) (White et al., 2014). Galectin-1 has emerged as a therapeutic target and reliable biomarker for a variety of tumors, which can be used to describe the clinical response and prognosis of patients. It is also expected to become a therapeutic target and prognostic marker for ccRCC. Some galectin-1 inhibitors have been developed for the treatment of tumors. For example, the Galectin-1 inhibitor showed significant anti-cancer effects both *in vitro* and *in vivo* in thyroid cancer lines expressing Gal-1 (Gheysen et al., 2021), and the Galectin-1 inhibitor had been used to treat B-cell precursor acute lymphoblastic leukemia (Paz et al., 2018) and head and neck squamous cell carcinomas (Koonce et al., 2017). Moreover, the Galectin-1 inhibitor combined with sorafenib was used in the treatment of liver cancer (Leung et al., 2019). The new strategy of targeting Galectin-1 in the treatment of ccRCC needs more research and is expected to improve the prognosis of ccRCC patients.

Nevertheless, there were some limitations in this study. Even if we have performed a lot of bioinformatics analysis to determine the potential diagnostic genes of ccRCC, the number of ccRCC samples obtainable in the public database is still limited, which may lead to potential errors/biases. In addition, the molecular mechanisms of the effects of LGALS1 on the prognosis and survival of patients with ccRCC need more experiments and verification on a larger scale.

In conclusion, we identified a hub gene, LGALS, by WGCNA and differential gene expression analysis in patients with ccRCC, which can serve as a potential biomarker for predicting prognosis and treatment targets. Our research provided new insights for exploring the prognosis and therapeutic targets of ccRCC patients.

## Data Availability

The original contributions presented in the study are included in the article/Supplementary Material, further inquiries can be directed to the corresponding author.
