# Supplementary material for: LGALS1 was related to the prognosis of clear cell renal cell carcinoma identified by weighted correlation gene network analysis combined with differential gene expression analysis
Source: Front Genet. 2023 Jan 12;13:1046164. doi: 10.3389/fgene.2022.1046164 (PMC9878452; doi:10.3389/fgene.2022.1046164)
Supplement: Supplementary file 5 [file Table1.DOCX]

|  |  |  |  |  |
| --- | --- | --- | --- | --- |
| **Table 1** Clinical data of TCGA-KIRC. | | | |  |
|  |  |  | Sample number | |
| Gender |  |  |  |  |
|  | Female |  | 191 |  |
|  | Male |  | 346 |  |
| Grade |  |  |  |  |
|  | G1 |  | 14 |  |
|  | G2 |  | 230 |  |
|  | G3 |  | 207 |  |
|  | G4 |  | 78 |  |
|  | GX |  | 5 |  |
|  | unknow |  | 3 |  |
| Stage | Stage I |  | 269 |  |
|  | Stage II |  | 57 |  |
|  | Stage III |  | 125 |  |
|  | Stage IV |  | 83 |  |
|  | unknow |  | 3 |  |
|  |  |  |  |  |
